# Rapid detection of ceftriaxone-resistant *Neisseria gonorrhoeae*: evaluation of HRM, LAMP, and qPCR targeting *penA*-60.001

**DOI:** 10.1128/spectrum.00514-26

**Published:** 2026-05-27

**Authors:** Yanhong Zhao, Linshan Dai, Lihua Hu, Mingqin Lu, Shaochun Chen, Faxing Jiang, Jia Huang

**Affiliations:** 1 School of Public Health, Nanjing Medical University 12461, Nanjing, China; 2 Department of Dermatology, Division of Life Sciences and Medicine, The First Affiliated Hospital of USTC, University of Science and Technology of China 12652, Hefei, China; 3 Bengbu Medical University 74539 , Bengbu, China; 4 Zhejiang Provincial Dermatology Hospital 561055 , Deqing, China; 5 Hospital for Skin Diseases, Institute of Dermatology, Chinese Academy of Medical Sciences & Peking Union Medical College, Nanjing, China; The University of Alabama at Birmingham, Birmingham, Alabama, USA

**Keywords:** *Neisseria gonorrhoeae*, ceftriaxone, molecular diagnostics, loop-mediated isothermal amplification, high-resolution melting

## Abstract

The global dissemination of ceftriaxone-resistant *Neisseria gonorrhoeae* threatens effective treatment and necessitates rapid diagnostic tools for surveillance. This study systematically evaluated three established molecular assays to identify practical and reliable approaches for detecting key resistance-associated *penA* markers, supporting evidence-based clinical decision-making. A total of 150 *N. gonorrhoeae* isolates were selected from an existing strain collection. Ceftriaxone minimum inhibitory concentrations (MICs) were determined by agar dilution, and full-length *penA* sequences were obtained by Sanger sequencing. High-resolution melting (HRM), loop-mediated isothermal amplification (LAMP), and real-time quantitative PCR (qPCR) were applied to genomic DNA from all isolates. Assay performance was assessed based on the detection of the A311V substitution, identification of the *penA*-60.001 allele, and classification of ceftriaxone-resistant isolates. Sanger sequencing revealed 34 distinct *penA* genotypes, confirming that 96 isolates carried the A311V substitution. While the dominant type was *penA*-60.001 (*n* = 90), this mutation was also found in other types, including *penA*-37.001 and *penA*-294.001. For detecting *penA*-60.001, HRM and LAMP assays demonstrated high accuracy (sensitivity above 98%, specificity over 91%), significantly outperforming qPCR. The assays also showed substantially higher specificity when directly detecting the A311V substitution across all *penA* types. However, when assessed against the ceftriaxone resistance phenotype, the sensitivity of all methods dropped to approximately 90%, due to the presence of resistant strains lacking the A311V mutation. Both HRM and LAMP reliably detect A311V and *penA*-60.001. Given its high accuracy, straightforward interpretation, and minimal infrastructure requirements, LAMP is particularly suitable for routine clinical diagnostics and antimicrobial resistance surveillance.

**IMPORTANCE:**

Rapid identification of antimicrobial-resistant *Neisseria gonorrhoeae* is essential for effective laboratory diagnostics and resistance surveillance. By systematically comparing three molecular assays across diverse *penA* genotypes, this study clarifies the strengths and limitations of commonly used approaches for detecting ceftriaxone resistance markers. The findings provide practical guidance for diagnostic laboratories seeking reliable and resource-appropriate methods to support antimicrobial resistance monitoring.

## INTRODUCTION

Gonorrhea, caused by *Neisseria gonorrhoeae*, is among the most common sexually transmitted infections (STIs), typically manifesting as inflammation of the urogenital tract. Without timely and effective treatment, it can lead to severe complications, including female infertility and an elevated risk of HIV acquisition and transmission (1, 2). Over recent decades, *N. gonorrhoeae* has progressively acquired resistance to multiple antimicrobial classes, including extended-spectrum cephalosporins and azithromycin (3). The emergence and international spread of resistant *N. gonorrhoeae* have been recognized by the World Health Organization (WHO) as a global public health threat (4). While ceftriaxone remains the first-line treatment for gonorrhea, resistant isolates have prompted guideline updates recommending higher doses (1, 5–7). Nevertheless, treatment failures are reported despite these adjustments, sometimes requiring retreatment with alternative antibiotics, including ertapenem (8–10). This increases healthcare burden and transmission risk, highlighting the need for timely identification of resistant strains at the initial visit.

The ceftriaxone-resistant strain FC428, first identified in Japan in 2015, carries the resistance determinant *penA*-60.001 and has since disseminated internationally (11). The A311V substitution in this mosaic *penA* allele is a key contributor to reduced ceftriaxone susceptibility in *N. gonorrhoeae*, and has recently been identified as a major molecular marker for ceftriaxone resistance detection (12). In recent years, various molecular assays have shown great promise for FC428 detection (13–18). Specifically, high-resolution melting (HRM), loop-mediated isothermal amplification (LAMP), and real-time quantitative PCR (qPCR)-based methods have been established and applied in previous studies (13–15). In this study, we systematically evaluated the three methods for their capacity to detect *penA*-60.001, the A311V mutation, and ceftriaxone resistance, providing evidence to support their clinical implementation.

## MATERIALS AND METHODS

### Isolates tested in the study

A total of 150 *N. gonorrhoeae* strains were selected from the existing isolate collection of archived clinical isolates obtained through routine diagnostic and surveillance activities across multiple regions in China between 2021 and 2023 to represent diverse *penA* genotypes. WHO reference strains P (*penA*-2.001), G (*penA*-2.001), and K (*penA*-10.001) were included as negative controls representing genetically diverse non-*penA*-60.001 backgrounds (including both non-mosaic and mosaic alleles without A311V), while China’s first FC428-like isolate BJ16148 (*penA*-60.001) was used as a positive control. Genomic DNA was extracted using the QIAamp DNA Mini Kit (Qiagen, USA) with the GeneRotex 96 Nucleic Acid Extractor (Tianlong, China). Sanger sequencing of *penA* was performed by Beijing Liuhe BGI Genomics Co., Ltd. (China). To identify the *penA* genotypes, all isolates were subjected to *N. gonorrhoeae* Sequence Typing for Antimicrobial Resistance analysis using the PubMLST database (https://pubmlst.org/) (19, 20).

### HRM, LAMP, and qPCR assays for detection of *penA*-60.001

The 150 isolates were tested for the *penA*-60.001 gene using established HRM, LAMP, and qPCR assays (13–15). All primers and the probe were synthesized commercially based on previously published protocols (Table 1) (13–15). Both the HRM and LAMP assays were developed to target the A311V substitution for detecting the *penA*-60.001 allele, while the qPCR assay was designed based on three A311V-positive alleles: *penA*-60.001, *penA*-59.001, and *penA*-64.001. Reactions were performed using the LightCycler 480 Instrument II (Roche, USA), and melting curve analysis was conducted with LightCycler 480 Software 1.5.1.

**TABLE 1:** Primers and probes used in HRM, LAMP, and qPCR assays in this study^*a*^

Methods	Primer and probe	Sequence (5′ → 3′)
HRM	*penA*-60.001-F	CCACTTTGCCTGAATCCAATGC
	*penA*-60.001-R	ACCGACATGATCGAACCTGG
LAMP	*penA-*60.001-F3	CAAAGACATCATCCTTTCCCTC
	*penA-*60.001-B3	GTGGGTATCTTGTACGGTATC
	*penA*-60.001-FIP (F1c+F2)	GCGGGCATCCAAAACCACCGAGGATTCAGACCTTGGCC
	*penA*-60.001-BIP (B1c+B2)	TCACGAAGCCGTTTACCATTGGGTATTGAATGTGTCTGATGG
	*penA*-60.001-FL	ACCGTTCCGGCTTTTGC
	*penA*-60.001-BL	GCCAAAGCATTGGATTCAG
qPCR	*penA*-60.001-F	CCCCGCGGACTCAAGCA
	*penA*-60.001-R	GTATTGAATGTGTCTGTTGGA
	Probe	FAM-ACCTGGTTCTGTCATGAAGCCGT-TAMRA

^*a*^ Published HRM and LAMP assays were originally designed with additional primers, including internal controls (*opa *and *porA*) and general *penA *primers, to ensure specificity against other *Neisseria species*. In this study, as all samples were confirmed *N. gonorrhoeae* isolates, general *penA *detection yielded uniformly positive results; therefore, only the *penA*-60.001-specific primer set was used for subsequent analysis.

The HRM assay differentiates DNA variants by analyzing the melting behavior of double-stranded DNA during gradual heating, generating distinct melting temperatures (Tm) and curves (21). In this study, each 20 µL reaction contained 10 µL of 2× Taq Pro HS U+ Probe Master Mix (Vazyme, China), 1 µL of 20× EvaGreen dye (Biotium, USA), 1 µL of each primer (10 µM), 2 µL of DNA template, and nuclease-free water to volume. Cycling conditions were 95°C for 10 min, followed by 30 cycles of 95°C for 15 s and 60°C for 1 min, and a final extension at 60°C for 1 min. HRM curves were obtained from 60°C to 95°C at 0.025°C/s. Samples were considered positive if their melting temperatures (Tms) and melting curves matched the *penA*-60.001 control; all others were considered negative.

The LAMP assay employs six primers that bind to multiple regions of the target gene, enabling efficient amplification, with results visualized through turbidity or fluorescence signals (22, 23). In this study, the LAMP assay was performed using the Fluorescent LAMP/RT-LAMP Kit (Vazyme, China). Each 20 μL reaction contained 12.5 μL of 2× Fluorescent LAMP/RT-LAMP Master Mix (Vazyme, China), 0.4 μL each of FIP and BIP primers (100 μM), 0.05 μL each of F3 and B3 primers (100 μM), 0.2 μL each of FL and BL primers (100 μM), 0.5 μL of 50× LAMP fluorescent dye (Vazyme, China), 2 μL of DNA template, and nuclease-free water to the final volume. Reactions were conducted at 60°C for 60 cycles (1 min per cycle), followed by melt curve analysis. The amplification results were monitored via fluorescence using the LightCycler 480 Instrument II (Roche, USA). Reactions with detectable fluorescence were considered positive; those without amplification were negative.

The qPCR assay was performed in a final volume of 20 µL, containing 10 µL of 2× Taq Master Mix (Dye Plus) (Vazyme, China), 0.8 µL of each primer (10 µM), 0.4 µL of probe (10 µM), 2 µL of DNA template, and nuclease-free water to volume. Cycling conditions were 95°C for 2 min, followed by 45 cycles of 95°C for 5 s and 60°C for 35 s. The results from the three assays were used to evaluate performance in identifying *penA*-60.001, detecting the A311V substitution, and predicting phenotypic ceftriaxone resistance. Amplification with Ct ≤ 40 was defined as positive; absence of amplification or Ct > 40 was negative.

### Antimicrobial susceptibility test

Ceftriaxone susceptibility was determined by agar dilution according to the WHO laboratory manual (24). Susceptibility categorization was based on European Committee on Antimicrobial Susceptibility Testing breakpoints adopted in the manual, with resistance defined as minimum inhibitory concentration (MIC) > 0.125 mg/L (24).

### Statistical analysis

Diagnostic performance, including sensitivity, specificity, overall accuracy, and corresponding 95% confidence intervals (CIs), was determined using the OpenEpi (http://www.openepi.com/), with Sanger sequencing results and antimicrobial resistance data serving as the reference standards.

## RESULTS

### *penA* profile of the isolates

Sanger sequencing identified 34 *penA* genotypes among the 150 isolates, including 30 known and 4 unclassified types. These comprised 8 mosaic genotypes (*n* = 107) and 22 non-mosaic genotypes (*n* = 39). The predominant mosaic genotype was *penA*-60.001 (*n* = 90). Among the four unclassified types, two differed from *penA*-60.001 by only a single nucleotide.

Amino acid alignment revealed the resistance-associated A311V substitution in *penA*-37.001, *penA*-60.001, *penA*-294.001, and three unclassified genotypes. Antimicrobial susceptibility testing (AST) confirmed that all these isolates carrying A311V were resistant to ceftriaxone. Conversely, among mosaic *penA* types lacking A311V, resistance was limited to a few isolates with *penA*-10.001, *penA*-34.006, and *penA*-34.019. In the non-mosaic group, resistance was observed in some isolates carrying *penA*-13.002, *penA*-19.001, *penA*-87.001, and *penA*-103.004 (Table 2).

**TABLE 2:** Summary of *penA* genotypes, antimicrobial resistance, MIC ranges, and ceftriaxone resistance-associated mutations in the strain panel (*n* = 150)

*penA* alleles	No. of isolates	Frequency (%)	Resistant isolates (%)^*a*^	Mutations related to ceftriaxone resistance^*b*^	MIC ranges (mg/L)
Mosaic *penA*					
Mosaic *penA* without A311V mutation					
10.001	7	4.5	33.3 (2/6)	I312M, V316T	≤0.008–0.5
34.001	1	0.6	0.0	I312M, V316T	≤0.008
34.006	2	1.3	50.0 (1/2)	I312M, V316T	≤0.008–0.5
34.007	2	1.3	0.0	I312M, V316T	0.015–0.06
34.019	2	1.3	50.0 (1/2)	I312M, V316T	0.03–0.25
Mosaic *penA* with A311V mutation					
37.001	1	0.6	100.0 (1/1)	A311V, I312M, V316P	>4
60.001	90	60.0	100.0 (83/83)	A311V, I312M, V316P	0.25 to >4
294.001	2	1.3	100.0 (2/2)	A311V, I312M, V316P	0.25
**Total mosaic *penA***	107	71.3	90.9 (90/99)	–	≤0.008 to >4
Non-mosaic *penA*					
2.001	2	1.3	0.0	None	≤0.008
2.002	4	2.6	0.0	None	≤0.008–0.03
5.002	3	1.9	0.0	None	0.015–0.03
5.014	1	0.6	0.0	None	≤0.008
5.016	1	0.6	0.0	None	0.06
12.001	1	0.6	0.0	None	0.015
13.001	1	0.6	0.0	None	0.03
13.002	3	1.9	33.3 (1/3)	None	≤0.008–0.25
17.001	3	1.9	0.0	None	0.06–0.125
18.001	3	1.9	0.0	None	0.015–0.125
19.001	2	1.3	50.0 (1/2)	None	≤0.008–0.25
21.001	3	1.9	0.0	None	≤0.008–0.03
22.001	2	1.3	0.0	None	≤0.008–0.015
43.001	1	0.6	0.0	None	≤0.008
43.002	1	0.6	0.0	None	0.06
43.004	1	0.6	0.0	None	≤0.008
44.001	1	0.6	0.0	None	≤0.008
87.001	1	0.6	100.0 (1/1)	None	0.25
103.001	2	1.3	0.0	None	≤0.008–0.03
103.004	1	0.6	100.0 (1/1)	None	0.25
120.001	1	0.6	0.0	None	≤0.008
187.001	1	0.6	0.0	None	≤0.008
**Total non-mosaic** ***penA***	39	26.0	10.3 (4/39)	–^*d*^	≤0.008–0.25
NA^*c*^	4	2.7	75.0 (3/4)	–^*d*^	0.015–1
**Total**	150	100	68.2 (97/142)	–^*d*^	≤0.008 to >4

^*a*^ MICs of 142 isolates were available for final evaluation.

^*b*^ Substitutions are limited to the A311V-containing region in *penA *(positions 311–316): A311V, I312M, V316P/T.

^*c*^ Four sequences do not match exactly in the PubMLST database.

^*d*^ “–” indicates that mutations could not be uniformly summarized.

### Evaluation of the HRM, LAMP, and qPCR assays among isolates

For the HRM assay, due to the use of a distinct amplification platform compared with the original report (13), Tm thresholds had to be established to ensure accurate interpretation. The resulting Tm profiles successfully distinguished the *penA* genotypes, yielding distinct ranges for A311V-positive mosaic *penA* (77.5°C–78.0°C), mosaic *penA* without A311V (78.5°C), and non-mosaic *penA* (79.0°C–79.5°C).

A total of 150 *N. gonorrhoeae* isolates were tested by HRM, LAMP, and qPCR, and the results were evaluated across three analytical dimensions reflecting different detection targets. For detecting the *penA*-60.001 allele, the LAMP assay yielded five false positives and one false negative. The HRM method yielded five false positive results and no false negatives. The qPCR method exhibited 14 false positives among 150 samples and no false negatives, leading to lower specificity. Overall, for *penA*-60.001 detection, both HRM and LAMP showed high sensitivity (>98%) and specificity (>90%), whereas qPCR had lower specificity (76.7%) despite maintaining 100% sensitivity (Table 3). Notably, the five false-positive results obtained with the HRM assay were identical to those observed with the LAMP assay. All five isolates carried *penA* alleles with the A311V mutation (two *penA*-294.001 and three unclassified *penA*) and were resistant to ceftriaxone (MICs = 0.25–1 mg/L).

**TABLE 3:** Evaluation results for HRM, LAMP, and qPCR

Detection targets	Methods	Sensitivity (95% CI), %	Specificity (95% CI), %	Overall accuracy (95% CI), %
*penA-*60.001 allele detection	HRM	100.0 (95.9–100.0)	91.7 (81.9–96.4)	96.7 (92.4–98.6)
	LAMP	98.9 (94.0–99.8)	91.7 (81.9–96.4)	96.0 (91.6–98.2)
	qPCR	100.0 (95.9–100.0)	76.7 (64.6–85.6)	90.7 (84.9–94.4)
A311V mutation detection	HRM	99.0 (94.3–99.8)	100.0 (93.4–100.0)	99.3 (96.3–99.9)
	LAMP	97.9 (92.7–99.4)	100.0 (93.4–100.0)	98.7 (95.3–99.6)
	qPCR	100.0 (96.2–100.0)	85.2 (73.4–92.3)	94.7 (89.8–97.3)
Ceftriaxone-resistant strain detection^*a*^	HRM	90.7 (83.3–95.0)	100.0 (92.1–100.0)	93.7 (88.4–96.6)
	LAMP	89.7 (82.1–94.3)	100.0 (92.1–100.0)	93.0 (87.5–96.1)
	qPCR	93.8 (87.2–97.1)	86.7 (73.8–93.7)	91.6 (85.8–95.1)

^*a*^ The strain panel included 150 isolates, and MICs were available for 142 isolates for final evaluation.

Targeting the A311V substitution, the HRM and LAMP assays achieved a sensitivity above 97% and 100% specificity (95% CI: 93.4%–100.0%), thus demonstrating superior accuracy compared to the qPCR method.

Furthermore, we assessed the capacity of each method to predict ceftriaxone resistance based on the AST results of the 142 isolates (8 of the original 150 isolates were excluded due to recovery failure during AST). When targeting resistant strains, all three methods showed reduced sensitivity compared to their performance in detecting *penA*-60.001 and the A311V substitution, with HRM and LAMP yielding 9 and 10 false negatives, respectively. These false-negative isolates carried a diverse range of *penA* alleles, including *penA*-10.001, *penA*-13.002, *penA*-19.001, *penA*-34.006, *penA*-34.019, *penA*-37.001, *penA*-87.001, and *penA*-103.004. Notably, except for *penA*-37.001, all of these *penA* types lacked the A311V substitution among their PBP2-associated resistance mutations (Table S1). Overall, the multi-target evaluation confirmed that all three methods achieved higher accuracy in detecting the A311V mutation compared to detection of the *penA*-60.001 allele and prediction of ceftriaxone resistance.

## DISCUSSION

Global reports of treatment failure due to ceftriaxone-resistant *N. gonorrhoeae* underscore the limitations of current regimens and the critical risk of ongoing transmission (8, 9, 25, 26). Furthermore, delays inherent in test-of-cure (TOC) procedures and subsequent retreatment can prolong infection and accelerate dissemination (9, 10, 25). Consequently, integrating molecular diagnostics into routine clinical practice is critical, as these assays can provide results 3–5 days earlier than conventional susceptibility testing, thereby facilitating more timely and targeted antimicrobial therapy.

In the original studies of three methods, both sensitivity and specificity for detecting *penA*-60.001 were reported as 100% (13–15). However, these initial evaluations were restricted to limited panels, typically including *penA*-60.001, *penA*-10.001, *penA*-34.001, and several WHO reference strains (with the HRM-based study not specifying the exact *penA* genotypes assessed). Given the known diversity of *penA* genotypes in clinical isolates, a more comprehensive evaluation panel is essential for reliable performance assessment. We addressed this need by selecting 150 isolates, which Sanger sequencing confirmed represented 34 distinct *penA* genotypes. This substantially broader genotypic range offers a more representative assessment of clinical diversity and provides a foundation for future research and clinical application. Because of potential cross-reactivity with other A311V-positive *penA* alleles, such as *penA*-294.001 and earlier variants like *penA*-59.001 and *penA*-64.001 reported before FC428 (27, 28), we evaluated each method for its ability to detect *penA*-60.001, the A311V mutation, and ceftriaxone-resistant strains.

Our findings indicate that the three assays exhibited lower specificity for detecting *penA*-60.001 compared to the A311V substitution itself. This reduced specificity is attributable to cross-reactivity with certain *penA* genotypes, such as *penA*-294.001 and three newly identified variants, which share identical mutations at positions 311–316 with *penA*-60.001, thereby generating false positives. Thus, A311V-based detection cannot reliably distinguish *penA*-60.001 from these rare genotypes. Notably, the continued emergence of A311V-positive strains, including *penA*-237.001 in France and the UK (29, 30), and *penA*-195.001 and *penA*-232.001 in China (18), highlights the potential for further spread of resistant lineages carrying this mutation. In this context, it is clinically more relevant to broaden the detection range of these assays to encompass all A311V-positive resistant strains, rather than restricting testing to FC428 or *penA*-60.001-related isolates. Additionally, a further limitation is incomplete coverage of A311V-positive isolates: for example, *penA*-37.001, which harbors A311V, was not detected by either HRM or LAMP. This is likely due to the distinct V316 substitution in *penA*-37.001 (V316P) compared to *penA*-60.001 (V316T), a difference that may affect primer binding in the LAMP assay, and possibly a nucleotide substitution at position 924 affecting the HRM assay.

The key amino acid substitution A311V in PBP2 is a major contributor to ceftriaxone resistance, yet other substitutions (I312M, V316P/T, T483S, A501V/P, N512Y, G542S, G545S, and P551S) are also associated with elevated ceftriaxone MICs (31–33). Accordingly, we evaluated the performance of these assays in detecting resistant isolates with diverse mutations, based on their ceftriaxone susceptibility. In contrast to detecting *penA*-60.001 or A311V mutations, the sensitivity of the assays decreased when detecting resistant strains, leading to false-negative results. These false-negative resistant strains carry eight *penA* genotypes, all lacking the A311V mutation except *penA*-37.001 (Table S1). This highlights the limitations of these assays in accurately identifying ceftriaxone-resistant *N. gonorrhoeae*. In the Netherlands, for example, predominant *penA* have shifted from mosaic to non-mosaic types with A501T/V (34). Consequently, local *penA* profiles should be considered when applying A311V-targeted detection methods. By contrast, in the Western Pacific region, ceftriaxone-resistant *N. gonorrhoeae* has risen substantially, especially strains with the *penA*-60.001 allele (35). Recent reports (2020–2023) indicate that this allele accounts for a substantial proportion of ceftriaxone-resistant strains in several regions of the Western Pacific (Table 4), including Hanoi, Vietnam (61%) (36), Hangzhou (45%), and Guangdong (39%), China (37, 38). Significantly, in Cambodia, *penA*-60.001 accounted for 93% (50/54) of ceftriaxone-resistant isolates reported from 2022 to 2023 (39). These findings suggest that assays targeting the *penA*-60.001 allele may serve as practical early-warning screening tools for ceftriaxone-resistant *N. gonorrhoeae* in regions where this lineage is prevalent, although they do not capture all resistance mechanisms.

**TABLE 4:** Reported prevalence of the *penA*-60.001 allele among ceftriaxone-resistant *N. gonorrhoeae* in selected areas of the Western Pacific region (2020–2023)

Region/city	Year	No. of resistant isolates^*a*^	No. with *penA*-60.001	Proportion^*b*^ (%)	Reference
Hangzhou, China	2020–2022	33	15	45	(38)
Guangdong, China	2021	31	12	39	(37)
Cambodia	2022–2023	54	50	93	(39)
Hanoi, Vietnam	2023	64	39	61	(36)

^*a*^ Different definitions of ceftriaxone resistance were used in the cited studies: references 31 and 33 defined resistance as MIC > 0.125 mg/L, whereas references 32 and 34 used MIC ≥ 0.125 mg/L.

^*b*^ Proportion refers to the percentage of *penA*-60.001-positive isolates among ceftriaxone-resistant isolates.

Beyond the A311V target, previous studies have developed a Six-Codon^plus^ HRM assay to identify additional mutations (A501, G542, and P551) (40, 41). After applying strategy 2 of this assay (positive defined as mosaic *penA* and non-mosaic alleles carrying A501 plus G542/P551 substitutions) to our samples (41), we achieved a high sensitivity of 97.9%, with only two false negative results (*penA*-19.001 and *penA*-103.004). However, this resulted in a relatively low specificity of 44.4%, with 25 false-positive results. This decline in specificity is due to strains carrying certain mosaic *penA* (*penA*-34.007) or other substitutions (A501, G542, and P551) that do not affect ceftriaxone susceptibility in this study. Therefore, while the Six-Codon^plus^ assay may be useful for preliminary screening, positive results still require confirmatory testing. These findings suggest that although multi-target strategies may improve sensitivity, their reduced specificity limits their feasibility as primary screening tools.

The three methods assessed in this study exhibit varying strengths concerning accuracy, detection time, result interpretation, and instrumentation requirements (Table 5). Across the three diagnostic evaluation targets in this study, HRM and LAMP showed comparable accuracy across detection targets, both outperforming qPCR. Regarding result interpretation, the LAMP assay is more straightforward, either by simple visual inspection or by fluorescence monitoring, requiring minimal training (23). While fluorescence-based detection was used for the LAMP assay in this study to ensure standardized evaluation, alternative readouts, such as visual colorimetric detection, can be easily applied in routine or low-resource settings, further reducing the technical skill required for data analysis (23). Conversely, the HRM method requires more complex data processing and interpretation of melting curves. Although the HRM method can further differentiate specific *penA* genotypes based on Tms, the small differences between Tm values can lead to misinterpretation by inexperienced personnel. To address this, we found that analyzing the post-melting transition point (Tpm) in normalized melting curves provides a clearer distinction between A311V-positive *penA* (78.5°C–79°C), mosaic *penA* without A311V (79.5°C), and non-mosaic *penA* (80.5°C). This analysis can serve as a valuable supplementary tool when interpreting complex melting curve data.

**TABLE 5:** The comparison of HRM, LAMP, and qPCR assays

	HRM	LAMP	qPCR
Performance^*a*^			
Sensitivity	High (90.7%–100.0%)	High (89.7%–97.9%)	High (93.8%–100.0%)
Specificity	High (91.7%–100.0%)	High (91.7%–100.0%)	Moderate to high (76.7%–86.7%)
Instrumentation requirement	Real-time PCR system (high-resolution melting)	Constant-temperature device (heating block/water bath)	Real-time PCR system
Interpretation difficulty	High (melting curve analysis)	Low (visual/fluorescence detection)	Moderate to high (Ct value analysis)
Reaction time (min)	72	60	32
Discrimination ability (mosaic/non-mosaic *penA*)	Yes (Tm/Tpm values)	No	No

^*a*^ Sensitivity and specificity ranges summarize performance across the three evaluation targets assessed in this study (*penA*-60.001 detection, A311V identification, and ceftriaxone resistance prediction).

In terms of instrumentation, the HRM assay requires more advanced thermal cyclers for precise temperature control and real-time fluorescence monitoring (21). While qPCR platforms are now widespread, the LAMP assay is the most accessible. Crucially, LAMP can be performed using simple constant-temperature heating devices, such as a water bath or heating block, without the need for thermocycling instruments, making it particularly suitable for primary laboratories and resource-limited settings (42). Therefore, the LAMP assay provides a practical option for antimicrobial resistance surveillance in remote settings without molecular diagnostic capacity, in line with the WHO’s strategy for equitable access to antimicrobial resistance containment tools (43). In terms of detection speed, the qPCR assay had the shortest reaction time (32 min), whereas the LAMP assay (60 min) and the HRM assay (72 min) both took over an hour. Importantly, although performed in batches on the LightCycler 480 in this study, the LAMP assay can also be applied to individual samples, supporting rapid single-patient testing.

Given its intuitive interpretation, lower instrumentation requirements, and high accuracy, the LAMP assay is more suitable for screening ceftriaxone-resistant *N. gonorrhoeae* in outpatient clinical settings. Results can be obtained within 2–3 h, enabling timely treatment decisions. A positive result suggests potential resistance and may warrant alternative regimens, such as dual therapy with azithromycin and gentamicin, or high-dose ceftriaxone (10), whereas a negative result should be interpreted in conjunction with standard diagnostic tests for *N. gonorrhoeae* and should not be used to exclude infection. Moreover, given the strong association between pharyngeal infections with resistant *N. gonorrhoeae* and treatment failure, pharyngeal sampling with LAMP testing is recommended at the initial clinical visit, particularly for patients with a history of oropharyngeal sexual contact (8, 9, 25). A positive initial test for resistant strains may also guide the rapid test during TOC. In summary, the molecular detection of resistant *N. gonorrhoeae* enables clinicians to make more targeted treatment decisions, thereby reducing the risk of treatment failure.

This study has several limitations. First, due to sample availability, only preserved *N. gonorrhoeae* isolates were tested, and further validation with clinical specimens will be important. Second, although previous studies have demonstrated high specificity of singleplex LAMP and HRM assays against other *Neisseria* spp., recent reports of *penA* sequences in non-gonococcal *Neisseria* closely resembling *penA*-60.001 raise concerns of potential cross-reactivity, warranting further evaluation (44).

### Conclusions

We evaluated three molecular assays for detecting ceftriaxone resistance-associated *penA* alleles. Considering ease of use and interpretation, LAMP is the most suitable for routine clinical implementation, while HRM offers additional value in distinguishing *penA* genotypes where suitable PCR platforms are available. However, all three assays are limited to A311V-associated alleles (*penA*-60.001, *penA*-294.001) and cannot fully capture the diversity of resistant *N. gonorrhoeae*. Expanding detection to additional loci (F504, A501, G542, P551) could improve coverage but requires balancing sensitivity with specificity. In conclusion, implementing the LAMP assay in STI clinical settings could provide essential technical support for global surveillance and precision treatment of resistant *N. gonorrhoeae*. Further optimization to maintain specificity while expanding detection to additional ceftriaxone resistance-associated loci will be a key direction for the next generation of resistance diagnostics.

## SUPPLEMENTAL MATERIAL

Table S1 spectrum.00514-26-s0001.docx penA alleles and mutations of false negative results from ceftriaxone resistance detection.

ASM does not own the copyrights to Supplemental Material that may be linked to, or accessed through, an article. The authors have granted ASM a non-exclusive, world-wide license to publish the Supplemental Material files. Please contact the corresponding author directly for reuse.

## References

[1] Workowski KA, Bachmann LH, Chan PA, Johnston CM, Muzny CA, Park I, Reno H, Zenilman JM, Bolan GA. 2021. Sexually transmitted infections treatment guidelines, 2021. MMWR Recomm Rep 70:1–187. doi:10.15585/mmwr.rr7004a1 PMC8344968 34292926

[2] Jones J, Weiss K, Mermin J, Dietz P, Rosenberg ES, Gift TL, Chesson H, Sullivan PS, Lyles C, Bernstein KT, Jenness SM. 2019. Proportion of incident human immunodeficiency virus cases among men who have sex with men attributable to gonorrhea and chlamydia: a modeling analysis. Sex Transm Dis 46:357–363. doi:10.1097/OLQ.0000000000000980 31095100 PMC6530490

[3] Sánchez-Busó L, Golparian D, Corander J, Grad YH, Ohnishi M, Flemming R, Parkhill J, Bentley SD, Unemo M, Harris SR. 2019. The impact of antimicrobials on gonococcal evolution. Nat Microbiol 4:1941–1950. doi:10.1038/s41564-019-0501-y 31358980 PMC6817357

[4] World Health Organization. 2024. WHO bacterial priority pathogens list, 2024: bacterial pathogens of public health importance, to guide research, development and strategies to prevent and control antimicrobial resistance. World Health Organization, Geneva.

[5] Unemo M, Ross J, Serwin AB, Gomberg M, Cusini M, Jensen JS. 2020. 2020 European guideline for the diagnosis and treatment of gonorrhoea in adults. Int J STD AIDS:956462420949126. doi:10.1177/0956462420949126 33121366

[6] Fifer H, Ismail MA, Soni S, Nwaosu U, Sadiq ST, Milligan A, Saunders J, Medland N. 2025. British association of sexual health and HIV UK national guideline for the management of infection with *Neisseria gonorrhoeae*, 2025. Int J STD AIDS 36:826–840. doi:10.1177/09564624251345195 40448704

[7] World Health Organization. 2024. Updated recommendations for the treatment of Neisseria Gonorrhoeae, Chlamydia Trachomatis, and Treponema Pallidum (Syphilis) and new recommendations on syphilis testing and partner services. World Health Organization, Geneva. 39137269

[8] Eyre DW, Sanderson ND, Lord E, Regisford-Reimmer N, Chau K, Barker L, Morgan M, Newnham R, Golparian D, Unemo M, Crook DW, Peto TE, Hughes G, Cole MJ, Fifer H, Edwards A, Andersson MI. 2018. Gonorrhoea treatment failure caused by a *Neisseria gonorrhoeae* strain with combined ceftriaxone and high-level azithromycin resistance, England, February 2018. Euro Surveill 23:1800323. doi:10.2807/1560-7917.ES.2018.23.27.1800323 29991383 PMC6152157

[9] Blouin K, Lefebvre B, Trudelle A, Defay F, Perrault-Sullivan G, Gnimatin JP, Labbé AC. 2024. *Neisseria gonorrhoeae* treatment failure to the recommended antibiotic regimen-Québec, Canada, 2015-19. J Antimicrob Chemother 79:3029–3040. doi:10.1093/jac/dkae327 39288011 PMC11531823

[10] Allan-Blitz LT, Fifer H, Klausner JD. 2024. Managing treatment failure in *Neisseria gonorrhoeae* infection: current guidelines and future directions. Lancet Infect Dis 24:e532–e538. doi:10.1016/S1473-3099(24)00001-X 38367636 PMC11391204

[11] Nakayama S-I, Shimuta K, Furubayashi K-I, Kawahata T, Unemo M, Ohnishi M. 2016. New ceftriaxone- and multidrug-resistant *Neisseria gonorrhoeae* strain with a novel mosaic penA gene isolated in Japan. Antimicrob Agents Chemother 60:4339–4341. doi:10.1128/AAC.00504-16 27067334 PMC4914677

[12] van Hal SJ, Lahra MM, Whiley DM. 2025. Determining targets for ceftriaxone resistance detection in *Neisseria gonorrhoeae* based on genotype-phenotype data: an observational study. Lancet Microbe 6:101199. doi:10.1016/j.lanmic.2025.101199 41075810

[13] Xiu L, Zhang C, Li Y, Wang F, Peng J. 2020. High-resolution melting analysis for rapid detection of the internationally spreading ceftriaxone-resistant *Neisseria gonorrhoeae* FC428 clone. J Antimicrob Chemother 75:106–109. doi:10.1093/jac/dkz395 31834402

[14] Shimuta K, Nakayama SI, Takahashi H, Ohnishi M. 2019. A loop-mediated isothermal amplification assay targeting *Neisseria gonorrhoeae penA*-60.001. Antimicrob Agents Chemother 64:penA–60. doi:10.1128/AAC.01663-19 PMC7187580 31658968

[15] Shimuta K, Igawa G, Yasuda M, Deguchi T, Nakayama SI, Ohnishi M. 2019. A real-time PCR assay for detecting a penA mutation associated with ceftriaxone resistance in *Neisseria gonorrhoeae*. J Glob Antimicrob Resist 19:46–49. doi:10.1016/j.jgar.2019.02.011 30825697

[16] Whiley DM, Mhango L, Jennison AV, Nimmo G, Lahra MM. 2018. Direct detection of penA gene associated with ceftriaxone-resistant *Neisseria gonorrhoeae* FC428 strain by using PCR. Emerg Infect Dis 24:1573–1575. doi:10.3201/eid2408.180295 30016236 PMC6056102

[17] Day MJ, Boampong D, Pitt R, Bari A, Rebec M, Saunders J, Fifer H, Mbisa JL, Cole MJ. 2024. Molecular detection of ceftriaxone resistance in *Neisseria gonorrhoeae* clinical specimens: a tool for public health control. Sex Transm Infect 100:454–456. doi:10.1136/sextrans-2024-056132 38925934

[18] Xiu L, Wang L, Li Y, Hu L, Huang J, Yong G, Wang Y, Cao W, Yang Y, Gu W, Peng J. 2025. Molecular screening to track ceftriaxone-resistant FC428-like *Neisseria gonorrhoeae* strains’ dissemination in four provinces of China, 2019 to 2021. Euro Surveill 30:2400166. doi:10.2807/1560-7917.ES.2025.30.6.2400166 39949323 PMC11914965

[19] Demczuk W, Sidhu S, Unemo M, Whiley DM, Allen VG, Dillon JR, Cole M, Seah C, Trembizki E, Trees DL, Kersh EN, Abrams AJ, de Vries HJC, van Dam AP, Medina I, Bharat A, Mulvey MR, Van Domselaar G, Martin I. 2017. *Neisseria gonorrhoeae* sequence typing for antimicrobial resistance, a novel antimicrobial resistance multilocus typing scheme for tracking global dissemination of *N. gonorrhoeae* strains. J Clin Microbiol 55:1454–1468. doi:10.1128/JCM.00100-17 28228492 PMC5405263

[20] Jolley KA, Bray JE, Maiden MCJ. 2018. Open-access bacterial population genomics: BIGSdb software, the PubMLST.org website and their applications. Wellcome Open Res 3:124. doi:10.12688/wellcomeopenres.14826.1 30345391 PMC6192448

[21] Reed GH, Kent JO, Wittwer CT. 2007. High-resolution DNA melting analysis for simple and efficient molecular diagnostics. Pharmacogenomics 8:597–608. doi:10.2217/14622416.8.6.597 17559349

[22] Notomi T, Okayama H, Masubuchi H, Yonekawa T, Watanabe K, Amino N, Hase T. 2000. Loop-mediated isothermal amplification of DNA. Nucleic Acids Res 28:E63. doi:10.1093/nar/28.12.e63 10871386 PMC102748

[23] Tomita N, Mori Y, Kanda H, Notomi T. 2008. Loop-mediated isothermal amplification (LAMP) of gene sequences and simple visual detection of products. Nat Protoc 3:877–882. doi:10.1038/nprot.2008.57 18451795

[24] World Health Organization. 2023. Laboratory and point-of-care diagnostic testing for sexually transmitted infections, including HIV. World Health Organization, Geneva.

[25] Fifer H, Natarajan U, Jones L, Alexander S, Hughes G, Golparian D, Unemo M. 2016. Failure of dual antimicrobial therapy in treatment of gonorrhea. N Engl J Med 374:2504–2506. doi:10.1056/NEJMc1512757 27332921

[26] Pleininger S, Indra A, Golparian D, Heger F, Schindler S, Jacobsson S, Heidler S, Unemo M. 2022. Extensively drug-resistant (XDR) *Neisseria gonorrhoeae* causing possible gonorrhoea treatment failure with ceftriaxone plus azithromycin in Austria, April 2022. Euro Surveill 27:2200455. doi:10.2807/1560-7917.ES.2022.27.24.2200455 35713023 PMC9205165

[27] Lahra MM, Ryder N, Whiley DM. 2014. A new multidrug-resistant strain of *Neisseria gonorrhoeae* in Australia. N Engl J Med 371:1850–1851. doi:10.1056/NEJMc1408109 25372111

[28] Deguchi T, Yasuda M, Hatazaki K, Kameyama K, Horie K, Kato T, Mizutani K, Seike K, Tsuchiya T, Yokoi S, Nakano M, Yoh M. 2016. New clinical strain of *Neisseria gonorrhoeae* with decreased susceptibility to Ceftriaxone, Japan. Emerg Infect Dis 22:142–144. doi:10.3201/eid2201.150868 26689442 PMC4696695

[29] Berçot B, Caméléna F, Mérimèche M, Jacobsson S, Sbaa G, Mainardis M, Valin C, Molina JM, Bébéar C, Chazelle E, Lot F, Golparian D, Unemo M. 2022. Ceftriaxone-resistant, multidrug-resistant *Neisseria gonorrhoeae* with a novel mosaic *penA-237.001* gene, France, June 2022. Euro Surveill 27:2200899. doi:10.2807/1560-7917.ES.2022.27.50.2200899 36695466 PMC9808317

[30] Day M, Pitt R, Mody N, Saunders J, Rai R, Nori A, Church H, Mensforth S, Corkin H, Jones J, Naicker P, Khan WM, Thomson Glover R, Mortimer K, Hylton C, Moss E, Pasvol TJ, Richardson A, Sun S, Woodford N, Mohammed H, Sinka K, Fifer H. 2022. Detection of 10 cases of ceftriaxone-resistant *Neisseria gonorrhoeae* in the United Kingdom, December 2021 to June 2022. Euro Surveill 27:2200803. doi:10.2807/1560-7917.ES.2022.27.46.2200803 36398578 PMC9673238

[31] Chen SC, Yin YP, Dai XQ, Unemo M, Chen XS. 2014. Antimicrobial resistance, genetic resistance determinants for ceftriaxone and molecular epidemiology of *Neisseria gonorrhoeae* isolates in Nanjing, China. J Antimicrob Chemother 69:2959–2965. doi:10.1093/jac/dku245 25011655

[32] Chen S-C, Yin Y-P, Dai X-Q, Unemo M, Chen X-S. 2016. First nationwide study regarding ceftriaxone resistance and molecular epidemiology of *Neisseria gonorrhoeae* in China. J Antimicrob Chemother 71:92–99. doi:10.1093/jac/dkv321 26472770

[33] Unemo M, Sánchez-Busó L, Golparian D, Jacobsson S, Shimuta K, Lan PT, Eyre DW, Cole M, Maatouk I, Wi T, Lahra MM. 2024. The novel 2024 WHO *Neisseria gonorrhoeae* reference strains for global quality assurance of laboratory investigations and superseded WHO *N. gonorrhoeae* reference strains-phenotypic, genetic and reference genome characterization. J Antimicrob Chemother 79:1885–1899. doi:10.1093/jac/dkae176 38889110 PMC11290888

[34] de Laat MM, Wind CM, Bruisten SM, Dierdorp M, de Vries HJC, Schim van der Loeff MF, van Dam AP. 2019. Ceftriaxone reduced susceptible *Neisseria gonorrhoeae* in the Netherlands, 2009 to 2017: from *PenA* Mosaicism to A501T/V Nonmosaicism. Sex Transm Dis 46:594–601. doi:10.1097/OLQ.0000000000001031 31415041

[35] Unemo Magnus, Lahra MM, Escher M, Eremin S, Cole MJ, Galarza P, Ndowa F, Martin I, Dillon J-AR, Galas M, Ramon-Pardo P, Weinstock H, Wi T. 2021. WHO global antimicrobial resistance surveillance for *Neisseria gonorrhoeae* 2017–18: a retrospective observational study. The Lancet Microbe 2:e627–e636. doi:10.1016/S2666-5247(21)00171-3 35544082

[36] Adamson PC, Hieu VN, Nhung PH, Whiley DM, Chau TM. 2024. Ceftriaxone resistance in *Neisseria gonorrhoeae* associated with the penA-60.001 allele in Hanoi, Viet Nam. Lancet Infect Dis 24:e351–e352. doi:10.1016/S1473-3099(24)00230-5 38723652 PMC11145020

[37] Liao Y, Xie Q, Yin X, Li X, Xie J, Wu X, Tang S, Liu M, Zeng L, Pan Y, Yang J, Feng Z, Qin X, Zheng H. 2024. penA profile of *Neisseria gonorrhoeae* in Guangdong, China: Novel penA alleles are related to decreased susceptibility to ceftriaxone or cefixime. Int J Antimicrob Agents 63:107101. doi:10.1016/j.ijantimicag.2024.107101 38325722

[38] Yang F, Sun X, Fu Y, Zhao F, Lin X, Chen Y, van der Veen S. 2024. High-level ceftriaxone resistance due to transfer of penA allele 60.001 into endemic gonococcal lineages in Hangzhou, China. J Antimicrob Chemother 79:2854–2857. doi:10.1093/jac/dkae297 39186249

[39] Ouk V, Say HL, Virak M, Deng S, Frankson R, McDonald R, Kersh EN, Wi T, Maatouk I, van Hal S, Lahra MM, EGASP Cambodia working group. 2024. World health organization enhanced gonococcal antimicrobial surveillance programme, Cambodia, 2023. Emerg Infect Dis 30:1493–1495. doi:10.3201/eid3007.240354 38916864 PMC11210631

[40] Li Y, Zhang L, Xiu L, Wang D, Zeng Y, Wang F, Yin Y, Peng J. 2022. A multiplex molecular assay for detection of six *penA* codons to predict decreased susceptibility to cephalosporins in *Neisseria gonorrhoeae*. Antimicrob Agents Chemother 66:e0170921. doi:10.1128/AAC.01709-21 35007131 PMC8923170

[41] Wang L, Li Y, Xiu L, Hu L, Huang J, Yong G, Wang Y, Cao W, Yang Y, Wang F, Gu W, Peng J. 2024. Multigeographic clinical assessment of a molecular diagnostic assay for detection of key codons to predict decreased susceptibility or resistance to cephalosporins in *Neisseria gonorrhoeae*. Antimicrob Agents Chemother 68:e0116524. doi:10.1128/aac.01165-24 39470197 PMC11620283

[42] Egbo TE, Blancett CD, Payne JM, Stefan CP, Minogue TD, Sellers JH, Koehler JW. 2025. Rapid identification of bacterial select agents using loop-mediated isothermal amplification. BMC Infect Dis 25:63. doi:10.1186/s12879-024-09573-w 39810089 PMC11734227

[43] World Health Organization. 2015. Global action plan on antimicrobial resistance. World Health Organization, Geneva.

[44] Shimuta K, Ohama Y, Ito S, Hoshina S, Takahashi H, Igawa G, Dorin Yamamoto M, Akeda Y, Ohnishi M. 2025. Emergence of ceftriaxone-resistant *Neisseria gonorrhoeae* through horizontal gene transfer among neisseria species. J Infect Dis 232:152–161. doi:10.1093/infdis/jiaf008 39776180

