# Supplementary material for: Rapid detection of ceftriaxone-resistant Neisseria gonorrhoeae: evaluation of HRM, LAMP, and qPCR targeting penA-60.001
Source: Microbiol Spectr. 2026 May 27;14(7):e00514-26. doi: 10.1128/spectrum.00514-26 (PMC13340278; doi:10.1128/spectrum.00514-26)
Supplement: Table S1 — penA alleles and mutations of false negative results from ceftriaxone resistance detection. [file spectrum.00514-26-s0001.docx]

**Supplementary data**

**Table S1** *penA* alleles and mutations of false negative results from ceftriaxone resistance detection

| *penA* | Mutations in PBP2 |
| --- | --- |
| 10.001 | I312M, V316T, N512Y, G545S |
| 13.002 | A501V, F504L, A517G, P551S |
| 19.001 | F504L, A517G |
| 34.006 | I312M, V316T, F504L, N512Y, G545S |
| 34.019 | I312M, V316T, F504L, N512Y, G545S |
| 37.001 | A311V, I312M, V316P, T483S, F504L, N512Y, G545S |
| 87.001 | A501T, F504L, A517G, P551S |
| 103.004 | A501V, F504L, A517G |
